# mpMRI-Based Risk Estimation to Optimize Prostate Cancer Patient Selection for Active Surveillance

**DOI:** 10.3390/cancers18050842

**Published:** 2026-03-05

**Authors:** Veronica Wallaengen, Evangelia I. Zacharaki, Mohammad Alhusseini, Adrian L. Breto, Isabella M. Kimbel, Nachiketh Soodana-Prakash, Ahmad Algohary, Noah Lowry, Isaac R. L. Xu, Pedro F. Freitas, Sandra M. Gaston, Rosa P. Castillo Acosta, Oleksandr N. Kryvenko, Chad R. Ritch, Bruno Nahar, Mark L. Gonzalgo, Dipen J. Parekh, Alan Pollack, Sanoj Punnen, Radka Stoyanova

**Affiliations:** 1 Department of Radiation Oncology, University of Miami Miller School of Medicine, Miami, FL 33136, USA; 2 Desai Sethi Urology Institute, University of Miami Miller School of Medicine, Miami, FL 33136, USA; 3 Sylvester Comprehensive Cancer Center, University of Miami Miller School of Medicine, Miami, FL 33136, USA; 4 Department of Radiology, University of Miami Miller School of Medicine, Miami, FL 33136, USA; 5 Department of Pathology & Laboratory Medicine, University of Miami Miller School of Medicine, Miami, FL 33136, USA

**Keywords:** prostate cancer, lesion detection, multiparametric MRI, deep learning, radiomics, active surveillance, risk modeling

## Abstract

**Background/Objectives:** Active surveillance (AS) has emerged as a safe alternative to primary therapy for low- and select intermediate-risk prostate cancer (PCa), but optimal patient selection and surveillance strategies remain challenging due to limited risk stratification tools enabling early detection of lesions with high potential for histopathological progression. This study presents an integrated method for predicting prostate cancer progression within 12 months, aiming to improve AS patient selection by categorizing patients into two risk groups: rapid progressors who would benefit from immediate treatment and slow progressors suitable for AS. **Methods**: The risk assessment platform combines convolutional neural networks for automatic segmentation of prostate and suspicious-for-cancer lesions on multiparametric MRI (mpMRI) with logistic regression to estimate progression risk. The networks were trained on annotated lesions from radical prostatectomy specimen mapped to mpMRI. The prediction model incorporated pre-biopsy clinical variables (age, PSA, PI-RADS) and MRI-derived intratumoral radiomic features from 163 participants of a prospective clinical trial, using histopathological progression within 12 months as endpoint. **Results:** The clinical-radiomics model achieved an AUC of 0.84 in distinguishing rapid from slow progressors, using non-invasive monitoring techniques. In an independent test set, the model significantly improved AS patient selection, increasing negative predictive value by 18.5% compared to current standard-of-care (*p* < 0.001). **Conclusions:** The risk assessment platform shows promise for use during annual follow-up visits to reliably differentiate suitable AS candidates with stable disease from PCa patients who are likely to experience early progression.

## Simple Summary

Prostate cancer is the second leading cause of cancer-related deaths in the United States; however, approximately half of newly diagnosed patients present with low-risk, indolent disease. Active surveillance (AS) offers an alternative to immediate treatment by closely monitoring patients through serial blood biomarkers, multiparametric MRI, and repeat biopsies. In a prospective AS trial with annual follow-up for disease progression, this study evaluated the added prognostic value of quantitative MRI features automatically extracted from AI-identified, cancer-suspicious regions. A prediction model trained on 163 patients achieved an ROC-AUC of 0.84 in distinguishing between individuals with high risk of rapid progressions and those with stable disease, demonstrating the potential of AI-derived imaging biomarkers to improve risk stratification and patient selection for active surveillance.

## 1. Introduction

About 250,000 men are diagnosed with prostate cancer (PCa) yearly in the US [1], and nearly half have low-risk disease and must decide whether to undergo treatment. Active surveillance (AS) has emerged as a safe alternative to immediate treatment and is the recommended disease management strategy for low-risk (Grade Group (GG) 1) patients [2] and favorable intermediate-risk patients [3], as it has been shown to often preserve quality of life (QoL) for over 5 years [4]. AS protocols typically include periodic monitoring of prostate-specific antigen (PSA) levels, as well as prostate multiparametric (mp)MRI, and are frequently incorporated into PCa management guidelines. Consequently, the percentage of US patients enrolled in AS has more than doubled in the past decade [5]. However, concerns about missing the window for cure remain, as emerging data reveal increased risk of metastasis caused by prolonged follow-ups [6]. Despite existing tools like the National Comprehensive Cancer Network (NCCN) [7] risk groups, there remains a need for accurate methods to risk stratify PCa patients, specifically to (i) avoid treatment delays by early identification of patients harboring lesions with high risk of histopathological progression, (ii) postpone treatment to preserve QoL for low-risk patients suitable for AS, and (iii) safely tailor the number and frequency of repeat biopsies.

The integration of mpMRI into fusion biopsy techniques has substantially improved PCa diagnosis, detecting 30% more high-risk cases than standard template biopsies [8]. The Prostate Imaging Reporting and Data System (PI-RADS) (current version PI-RADSv.2.1) [9] has standardized reporting of suspicious prostate lesions; however, its semi-quantitative scoring shows low inter-reader agreement (<50%) and suboptimal interpretation [10]. Various computer-aided diagnosis and artificial intelligence (AI) techniques for mpMRI analysis have been implemented [11], harnessing the wealth of quantitative information in the multiple mpMRI sequences, typically with the objective of cancer detection and/or assessment of aggressiveness and focusing on clinically significant cancer (GG ≥ 2) [12]. Yet, MRI-based AI models focusing on AS management remain limited [13], with a recent review noting insufficient evidence to support implementation [14].

Here we present an integrated mpMRI-based platform that combines pre-biopsy imaging and clinical features from a prospective clinical trial where AS patients are monitored yearly with mpMRI and MRI-Ultrasound fusion (MRI-US) biopsies to predict the risk of early histopathological progression. Non-invasive risk monitoring over time may help identify optimal times for intervention or biopsy, thereby reducing poor outcomes from delayed primary treatment. The radiomic features selected for the model were found to significantly impact progression risk assessment when evaluated individually and to substantially increase model performance when combined with clinical variables. The model was also tested for prediction of progression within 12 months in an independent dataset and shown to improve AS patient selection compared to current standard-of-care.

## 2. Materials and Methods

### 2.1. Overview of Model Development and Implementation

The progression risk prediction model utilized data from MRI-Guided Active Selection for Treatment of Prostate Cancer: The Miami MAST Trial (MAST). The MAST study protocol entailed: an mpMRI and MRI-US biopsy (confirmatory) within 18 months of diagnosis, followed by surveillance biopsies at 12, 24, and 36 months, or until histopathologic progression occurred. Histopathological progression was defined as one or more of the following: (i) more than 4 cores with any grade cancer, (ii) more than 2 cores with GG2 cancer, (iii) any single core with GG3 or higher cancer, (iv) a GG1 at diagnosis upgraded to GG2 or higher. Patients who declined participation or decided to undergo treatment without histopathological progression before their first surveillance visit (approximately 12 months after their confirmatory visit) were excluded from the analysis. Two groups were identified among the MAST participants:

- Rapid progressors: participants who progressed within 12 months of their confirmatory, baseline visit.
- Slow progressors: participants who progressed at 24 or 36 months of their baseline visit or who completed the full trial without signs of histopathological progression.

**Figure 1:**
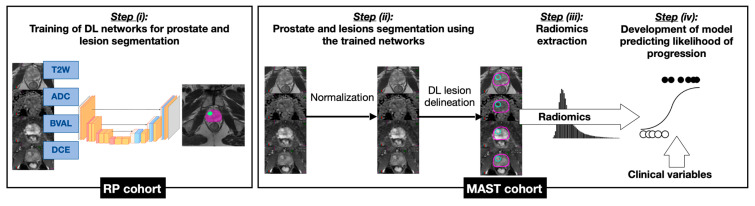
Overview of the risk estimation pipeline development.

A model predicting likelihood of histopathological progression within 12 months (MHP-12mo) was trained to distinguish between these two groups. Figure 1 depicts the development of MHP-12mo. Prostate and suspicious lesions were segmented using deep learning (DL) networks (Step (i)). 3D nnU-Net [15] networks were trained on mpMRI scans from patients who underwent radical prostatectomy (RP). Imaging data from MAST patients were then segmented (Step (ii)), and radiomic features were extracted from tumor lesions (Step (iii)). In addition to radiomic features, pre-biopsy clinical variables were also incorporated in the model (Step (iv)). MHP-12mo stratifies patients into those for whom annual mpMRI monitoring is recommended and those for whom definitive treatment (RP or radiotherapy) should be considered. The performance of MHP-12mo was evaluated in an independent set of 16 MAST participants who showed histopathological progression on their 24- or 36-month surveillance exam.

### 2.2. Development of Deep Learning Segmentation Networks for Prostate and Prostate Lesions on mpMRI

MpMRI data from 45 patients who have undergone radical prostatectomy (RP) were used for the training of DL networks for automatic segmentation of prostate and suspicious-for-cancer lesions. The University of Miami Institutional Review Board (IRB) approved a protocol for retrospective review of imaging from patients who underwent RP between December 2014 and September 2016 and had mpMRI on 3T MRI instruments and waived the need for informed consent. The MRIs were manually annotated with prostate contours by imaging experts. Tumor lesions were mapped from histological images onto MRI images using the approach described in Tschudi et al. [16,17]. In short, all tissue specimens were reviewed by a urologic pathologist (ONK) and cancerous areas were outlined and graded according to the latest recommendations [18,19] on hematoxylin and eosin (H&E) stained slides. The annotated slides were scanned, and prostate quadrants were “stitched” into pseudo whole-mount RP samples, which were used to map the lesion locations onto MRI in MIM (Cleveland, OH).

The DL network training data comprised the following transversal MR images: T2w, ADC, BVAL, and early enhancing series from the DCE-MRI. All MR images were first resampled with the T2w as reference to 0.5 mm x 0.5 mm axial spacing with the original z-spacing preserved. T2w intensities were normalized using a multi-reference normalization approach [20], where DL was used to segment three reference structures (gluteus maximus, femoral head and bladder) on a per-patient basis and a linear function fitted between the average intensities of these structures and fixed reference values. DCE and BVAL images were normalized by the average gluteus maximus intensity.

Two separate 4-channel 3D nnU-Net [15] convolutional neural networks were trained for segmentation of (1) prostate and (2) suspicious-for-cancer lesions. This type of network was selected due to its ability to automatically configure itself covering the entire segmentation pipeline, including data processing, network architecture and training procedure. To confine the lesion detections to the prostate region, the training images introduced to the lesion segmentation network were first cropped according to a bounding box identified around the manually contoured prostate. Each network was trained with deep supervision through 5-fold cross-validation using the standard nnU-Net configuration, and data augmentation was applied to avoid overfitting and increase the robustness of the network. The nnU-Net was implemented in Python using PyTorch version 1.13.0 as DL framework.

### 2.3. The Miami MAST Trial Multiparametric MRI and Biopsy Protocol

The Miami MAST Trial (ClinicalTrials.gov: NCT02242773) is a prospective, single-center, single-arm trial for men undergoing AS, approved by the IRB in 2014. The trial enrolled patients aged 35–85 years with PSA ≤ 20 ng/mL and biopsy-confirmed PCa. To satisfy enrollment criteria, diagnostic biopsies must have had a minimum of eight cores with maximum four positive cores and maximum two cores of GG2 cancer and be centrally reviewed by the study pathologist (ONK).

The confirmatory and subsequent surveillance mpMRI exams consisted of triplanar T2w MRI of the male pelvis, DWI and the associated ADC map, and DCE-MRI. MpMRI data were acquired on 3T Discovery MR750 (GE Healthcare, Waukesha, WI, USA), 3T Magnetom Skyra, TrioTim, Vida and 1.5T Magnetom Avanto (Siemens Healthineers, Erlangen, Germany) scanners at four different institutes within the University of Miami Health System using imaging sequences and sequence parameters consistent with PI-RADSv2.1 recommendations [21]. Imaging acquisition details are given in Supplementary Material, Table S1.

MpMRIs were reviewed and graded using PI-RADSv2.1 by one out of five radiologists with 2–20 years of experience in genitourinary malignancies. Prostate and suspicious-for-cancer regions were outlined in Dynacad (InVivo, Gainsville, FL, USA). Systematic 12-core template biopsies and targeted MRI-US fusion biopsies for PI-RADS3+ lesions were performed in UroNav (InVivo, Gainsville, FL, USA).

### 2.4. Radiomic Feature Extraction and Selection

A series of mpMRI-derived radiomic features were extracted from the DL-identified lesions, or the whole segmented prostate volume if no lesions were detected: intensity mean, minimum, maximum, standard deviation, skewness, kurtosis and 10th, 25th, 50th, 75th, and 90th percentiles from the ADC and normalized T2w, BVAL and DCE [22]. In addition, segmented prostate volume, largest lesion volume, combined volume of all identified lesions, and number of identified lesions were included as features.

The Minimum Redundancy-Maximum Relevance [23] feature selection method was applied to create a set of 12 top-ranked features. Cross-validated exhaustive feature search was then performed to determine the best combination between 3 and 12 of these features with respect to optimizing the AUC for prediction of histopathological progression using logistic regression. To ensure robust resampling and prevent data leakage, feature selection was performed strictly within the inner loop of a nested cross-validation scheme. In addition to the radiomic features, three clinical variables were also introduced as additional predictors indicative of progression: age, PSA level and highest PI-RADSv2.1 assessment category. Feature standardization was performed through Z-score normalization prior to model training.

For explainability, the predictive power of each selected feature was studied separately via box plots and differences in feature mean values between rapid and slow progressors were assessed via *t*-test statistics. In addition, Cox proportional hazard regression [24] and Kaplan–Meier survival analysis [25] were performed using maximally selected rank statistics [26] (MSRS) to find optimal cut-points for identifying high- and low-risk groups for each covariate. Differences in progression-free survival probability between the groups were assessed via log-rank test statistics.

Study data were collected and managed using REDCap electronic data capture tools [27] hosted at the University of Miami and extracted for the analysis, which was performed in Python 3.10.

### 2.5. Modeling of Progression Risk and Performance Evaluation

MHP-12mo, a logistic regression model utilizing the clinical-radiomics variables, was developed to assess the risk of histopathological PCa progression within the timeframe of a patient’s next surveillance visit by classifying patients into rapid and slow progressors. Due to the dataset size, Leave-One-Out Cross-Validation [28] was used for model training and validation, with 95% confidence intervals computed using patient-level bootstrapping (1000 bootstrap replicates). Improvements in negative predictive value (NPV) were assessed using the relative predictive value [29].

## 3. Results

### 3.1. Prostate and Lesion Segmentation

**Figure 2:**
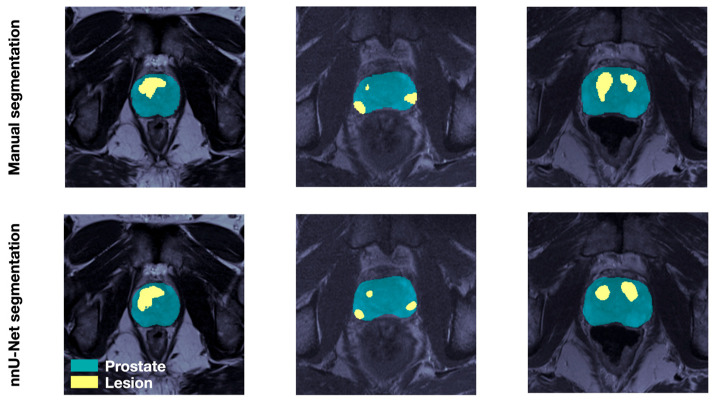
Results of nnU-Net segmentation. Prostate (cyan) manually segmented by experienced imaging experts and lesions (yellow) mapped from histology slides post RP (**upper**), as well as prostate and lesions segmented by the trained nnU-Net (**lower**) on corresponding T2w slides for three patients in the RP dataset.

The prostate segmentation mean Dice Similarity Coefficient (DSC) [30] was 0.84. The lesion detection performance was assessed with respect to expert delineations using the DSC and criteria defined in recent work of others [31,32], namely, (i) only dominant index lesions are examined, defined as the largest lesion volume with Gleason score > 6; (ii) DSC is computed for all lesions overlapping with manual segmentations, with the overlap defined as DSC > 0.1; and (iii) segmented lesion volumes under 0.1 cm^3^ are considered negligible. According to these criteria, the lesion segmentation mean and median DSC across the five training folds were 0.57 and 0.61 respectively. When considering all lesions, the lesion segmentation mean DSC was 0.43. Figure 2 illustrates the visual agreement between manual and DL-generated prostate and lesion masks for three select RP patients.

### 3.2. The Miami MAST Trial

**Figure 3:**
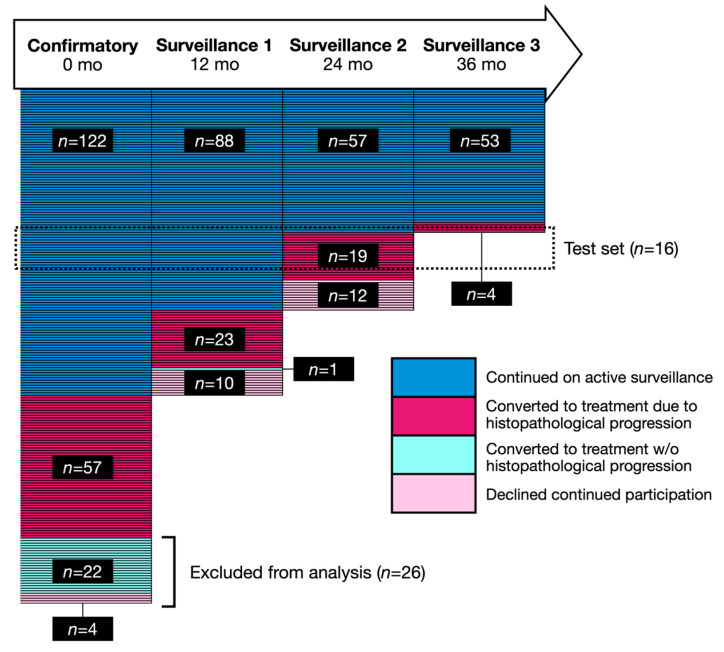
MAST timeline. Each horizontal line represents a study participant.

Two hundred and five patients were enrolled in MAST between 2014 and 2020. Figure 3 illustrates the patient enrollment and follow-up, with each row representing one participant. Twenty-six participants were excluded from the analysis: 22 withdrew from the study and four progressed to treatment without signs of histopathological progression before their first follow-up visit. Table 1 lists the clinical characteristics of the patients in the training set (*n* = 163) and test set (*n* = 16). Patients were classified as benign if all cores at the Confirmatory biopsy showed no evidence of cancer. Note that patients in the test set had significantly lower disease burden at confirmatory biopsy. This is because the test cohort consisted of patients who progressed on 24- or 36-month surveillance biopsy. Consequently, these patients did not meet the criteria for histopathological progression at confirmatory biopsy and were therefore predominantly classified as GG1 at baseline. This selection reflects the study design rather than sampling bias and explains the difference in grade distribution between the training and test cohorts.

### 3.3. Segmentation of Lesions in MAST Participants

**Figure 4:**
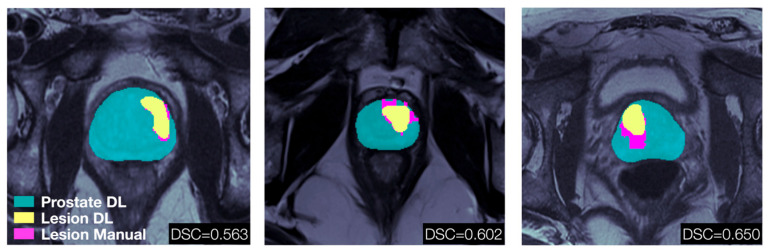
Results of applying the trained segmentation networks to MAST mpMRI data. DL-generated prostate masks (cyan) and lesion masks (yellow), as well as lesion masks manually outlined by an experienced radiologist and matched to biopsy results to confirm PCa (magenta), superimposed on T2w axial slices for three selected MAST participants. The stated DSC refers to the total lesion volume of each case, not the individual slices displayed.

The trained segmentation networks were used to automatically identify prostate and tumor lesions on confirmatory mpMRI exams of the MAST participants. A detected lesion was defined as any segmented volume ≥ 0.1 cm^3^. At least one lesion was detected in 91.1% of the analyzed patients. The median number of detected lesions in the MAST cohort were 2 (IQR: 1–2), which can be compared to the confirmed number of lesions in the RP cohort with a median of 4 (IQR: 2–5). This is expected as the MAST patients have less advanced disease compared to patients undergoing RP. In Figure 4, the prostate and lesion masks generated by the nnU-Net superimposed on T2w axial slices for three of the MAST participants are shown. The results are compared to prostate and lesions contours manually outlined by a radiologist with confirmed cancerous pathology from a subsequent biopsy for the same T2w image slices. The total lesion volume DSC of the selected samples ranged between 0.56 and 0.65 when considering all lesions.

Comparing the DL segmented prostate volume to the mpMRI estimate using the ellipsoid formula of the PI-RADS v2.1 guidelines [9], the median percentage difference was 11.3% (IQR: 5.3–20.6%). The median prostate volume of the mpMRI estimate and the DL segmentation was 44.1 mL and 40.0 mL respectively. A test of the Pearson correlation between the two volume distributions resulted in *r* = 0.92, *p* < 0.001. The median prostate volume was slightly higher when estimated using the PI-RADS v2.1 guidelines compared to the nnU-Net segmentation, in line with previous studies [33] showing that MRI tends to slightly overestimate prostate volume.

### 3.4. Radiomics and Model Predicting Likelihood of Histopathological Progression Within 12 Months (MHP-12mo)

**Figure 5:**
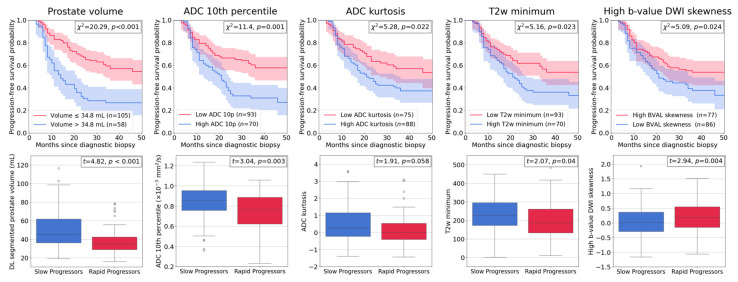
Results of individual evaluation of the five DL-based and radiomics intensity features selected for the progression risk prediction model MHP-12mo. (**Upper row**) Kaplan–Meier curves for each covariate with the patient population stratified into a faster progressing cohort shown in red and slower progressing cohort shown in blue, both with corresponding confidence intervals. The cohort stratification was determined by maximally selected rank statistics (MSRS) of feature values. Differences in progression-free survival probability between the two groups were assessed via log-rank test statistics (χ^2^). (**Bottom row**) Box plots displaying feature distributions of slow progressors (*n* = 83) in blue and rapid progressors (*n* = 80) in red. Differences in feature mean values between the two groups was assessed via *t*-test statistics (*t*).

Image intensity-based radiomic features were extracted from the normalized images. Among 48 evaluated features, Apparent Diffusion Coefficient (ADC) intensity 10th percentile and kurtosis, T2-weighted (T2w) intensity minimum, and intensity skewness of the Diffusion-Weighted Imaging (DWI) highest b-value sequence (BVAL) as well as the DL-segmented prostate volume were selected for the progression risk prediction model MHP-12mo. Computed hazard ratios for these features confirmed all to be significant risk factors for rapid progression (Table 2). Kaplan–Meier curves and box plots strengthened these findings (Figure 5). Lower ADC and T2w values as well as higher b-value DWI skewness were associated with higher risk of rapid progression, while smaller prostate volume also correlates with increased progression risk. Similar details of the three clinical predictors variables (PI-RADS, PSA, age) incorporated into the model are provided in Appendix A.

**Figure 6:**
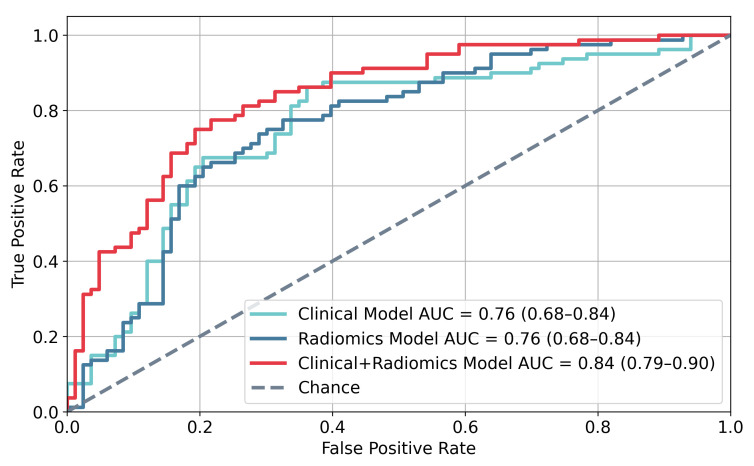
Receiver Operating Characteristic (ROC) curves of logistic regression models predicting time of histopathological progression (*n* = 163) when trained using clinical variables only (turquoise), radiomic features only (blue) and a combination of clinical variables and radiomic features (red). Area under the curve (AUC) is provided for each model with corresponding 95% confidence intervals in parenthesis.

MHP-12mo was built from eight clinical-radiomic features and trained on a 163 patient cohort (80 rapid progressors, 83 slow progressors) using progression within 12 months of the confirmatory exam as outcome. The accuracy, sensitivity, specificity, positive predictive value (PPV) and NPV in identifying rapid progressors were 78%, 77%, 78%, 77% and 78%, respectively. The model achieved an Area Under the Receiver Operating Characteristic Curve (AUC) of 0.84, which can be compared to 0.76 for an equivalent model trained only on clinical variables, as shown in Figure 6. A permutation test demonstrated the difference in AUC between the models to be statistically significant (*p* = 0.02).

### 3.5. Evaluation of MHP-12mo on Test Set

The test set comprised 16 MAST participants who showed histopathological progression on their 24- or 36-month surveillance exam (Table 3**,** second column). In Kimbel et al. [34] an imaging review using the quantitative Habitat Risk Score (HRS) [17] approach showed evidence of undetected histopathological progression prior to the second or third surveillance biopsy in 44% (7/16) of these patients. Reasons for this were suggested as either PI-RADS v2.1 missing the dominant lesions or biopsy sampling errors whereby the targeted lesion or the most aggressive part of the tumor was missed by the biopsy needle. In Table 3 (third column), the adjusted time of progression from this imaging re-evaluation is listed. For example, Patient #3 progressed at 36 months while the analysis in Kimbel et al. [34] showed that progression occurred earlier (12 months). MHP-12mo correctly identified this patient as a rapid progressor. In this case, application of the model would have saved two years of unnecessary AS. Using the Adjusted time of progression as ground truth for evaluating MHP-12mo, the model correctly classified 10 out of 16 patients. In contrast, current standard-of-care protocols labeled all 16 participants as slow progressors and none of the seven high-risk rapid progressors were identified as such. MHP-12mo improved AS patient selection by increasing the NPV of identifying rapid progressors by 18.5% (*p* < 0.001), from 56.3% (9/16) to 66.7% (6/9). Improvements in sensitivity, specificity, and PPV could not be meaningfully assessed as standard-of-care classified no participants of the test set as rapid progressors (no positive detections). Meanwhile, out of the seven participants who would benefit from immediate treatment instead of AS, MHP-12mo labeled four correctly; reducing 6 years of unnecessary AS and providing correct recommendations for conversion to treatment.

## 4. Discussion

Here we present an mpMRI-based risk estimation model that combines pre-biopsy imaging and clinical features: model predicting likelihood of histopathological progression within 12 months, MHP-12mo. By integrating multiple pipelines for prostate lesion segmentation and radiomics extraction, the model achieved AUC = 0.84 in differentiating suitable AS candidates with stable disease from patients who are likely to progress rapidly. Intratumoral radiomic features were found to add substantial value to the classification performance, confirming the hypothesis that imaging biomarkers play an important role in progression risk assessment. Additionally, MHP-12mo significantly improved PCa risk predictions in an independent patient cohort compared to current standard-of-care protocols, thereby demonstrating promising potential in improving AS patient selection and reducing treatment delays.

The performance of the network for prostate segmentation was comparable to the previously reported DSC values from our group and others [35,36,37]. Performance deteriorated for lesion segmentation, consistent with the known limitations of DSC when evaluating small structures, where even single-pixel variations can substantially affect the score [38]. Segmentation uncertainty at the lesion level may propagate into downstream radiomic feature extraction, particularly for features that are highly sensitive to boundary definition, such as shape descriptors and higher-order texture metrics. However, as our analysis was restricted to first-order intensity-based features, which are generally less dependent on precise boundary delineation, we expect the impact of segmentation variability on our radiomic measurements to be relatively limited. While the computed DSC is significantly higher when accounting only for index lesions harboring clinically significant PCa, the network is also able to identify low-grade tumor lesions, which makes it ideal for segmentation of mpMRI data from AS patients. Various similar networks have been trained to detect lesions that are visible to a human expert but may not be sensitive enough to identify low-grade cancer foci. The RP training dataset offers a key advantage over widely used MRI datasets annotated solely with radiologist contours: it provides better lesion localization, making it uniquely valuable for enhancing segmentation confidence. Apart from constituting an integral part of the risk estimation pipeline, the segmentation masks can potentially also be utilized to target lesions during surveillance biopsies. Further training of the nnU-Net with more data is considered important for improved performance.

Previous work on radiomics has been limited by the use of agnostic features lacking clear biological interpretation, high susceptibility to variations in scan protocol, and increased risk of false discovery and overfitting. Focusing on first-order features in our pipeline improves interpretability and clinical relevance. For example, two features selected for our model, “T2w minimum” and “ADC 10th percentile”, are directly related to lesion aggressiveness (Figure 5). The “T2w minimum” signal was lower in rapid progressors, consistent with previous reports linking lower T2w intensity to more aggressive prostate cancer [20]. Similarly, rapid progressors tended to have lower “ADC 10th percentile” values, reflecting restricted diffusion commonly associated with higher tumor cellularity. ADC values are inversely proportional to tumor cellularity and hypointense areas correspond to malignant tissue [39]. Overall, these observations are in line with the potential biological significance of the selected features.

MHP-12mo achieved sensible AUC performance, on par with other methods for predicting PCa progression [40]. Including intratumoral radiomic features significantly improved the AUC of the model compared to including clinical variables only. When evaluated separately, a prediction model trained only on DL-based quantities and radiomic features achieved the same AUC as a model trained only on clinical variables, indicating that the radiomic features identified in this study carry similar predictive power to clinical variables well-known to be associated with PCa progression. On the test set, MHP-12mo correctly identified 4/7 AS patients at high risk of rapid histopathological progression. These results suggest potential to reduce treatment delays by flagging patients for further evaluation who might otherwise have undetected progression during monitoring, but indicate that the current model alone is insufficient to determine histopathological progression risk. As such, the model is intended to guide exploratory analyses and support clinical reasoning rather than to serve as a definitive clinical tool. Its performance and generalizability need to be confirmed in larger, multicenter studies before broader adoption. With future improvements, the risk estimation platform could improve clinicians’ confidence in recommending AS as the preferred PCa management strategy for well-selected patients. Perhaps more importantly, the platform may help identify patients who can safely avoid biopsies and remain on AS using monitoring through imaging only.

This study introduces several novel elements, primarily the integration of multiple pipelines—DL-based prostate and lesion segmentation, normalization, radiomics extraction, and risk modeling—into a single framework. The use of histopathological progression as endpoint is also distinctive, as most imaging-based machine learning models focus on GG alone as the primary outcome. By modeling this endpoint, our analysis is directly aligned with clinically actionable risk assessment. Importantly, the prediction of histopathological progression within 12 months is clinically actionable, as it relies solely on non-invasive imaging and routinely available clinical features (PI-RADS, PSA, age), enabling patient-centered recommendations such as extending imaging intervals for individuals with low predicted risk or expediting biopsy and treatment for those at higher risk of rapid progression. Compared to a similar model [41] which used single-center, single-scanner baseline data to predict progression from radiomics extracted from manually segmented lesions, our approach demonstrates superior performance while also offering a higher degree of automation and being applicable to more heterogeneous data. Further, using histopathological progression as an outcome variable and leveraging data from a clinical trial with standardized inclusion criteria, follow-up schedule, and outcome definitions helps mitigate heterogeneity of AS protocols—a major barrier for the transition to AI-based AS which was characterized as “unrealistic” in a recent review of prostate MRI and AS [14].

In addition to high susceptibility to variations in scan protocols, previous work on radiomics has also been hampered by increased risk of false discovery and overfitting, as well as the use of agnostic features without clear meaning [42,43,44]. In this study, the selected imaging features were instead highly explainable and robust, considering the heterogeneous imaging data sources. The T2w minimum signal is of lower intensity among rapid progressors and this agrees with indications of more aggressive PCa [45]. Rapid progressors tended to have lower values of the ADC 10th percentile as well as lower values of ADC kurtosis, indicating that the lowest intensities are typically lower and the intensity data more evenly spread than in slow progressors. This is also the case for skewness of the BVAL, which signifies a higher degree of asymmetry in the intensity distribution of high-risk patients, with extreme values being more frequent on the higher end of the intensity spectrum than on the lower. These results indicate that the imaging of rapid progressors typically include bright appearing areas on the DWI at high b-values while more dark appearing areas are present on the ADC maps. This correlates with more aggressive disease as DWI at high b-values suppresses healthy background tissue and hyperintense areas reflect water molecules within the tissue having restricted movement, something that is associated with cancerous tissue density [46]; conversely, ADC values are inversely proportional to tumor cellularity and hypointense areas correspond to malignant tissue [39]. Another feature selected for the model was the prostate volume, which is known to be inversely related to prostate cancer aggressiveness [47]. Appendix A further elaborates on the explainability of the clinical predictor variables.

This study has several limitations. This is a single-center study, and therefore multicenter validation is necessary. Apart from discordance in non-standardized AS monitoring protocols [48], clinical applications utilizing radiomics models in AS are limited by the poor reproducibility of MRI-derived features [49] and image pre-processing variations [50]. The model was evaluated in a small prospective cohort, which introduces inherent limitations and raises concerns regarding generalizability to broader populations. Further, using histopathological progression as prediction outcome is challenging, as the criteria for when progression has been achieved is subject to uncertainty, as well as being prone to biopsy sampling errors. Re-evaluation of the test set data was performed to better assess the true time of progression; nevertheless, patients who did not progress over the course of the study period were not reviewed. Such a review may reveal more cases of missed progressors, which would impact the reliability of the model. While we focused on histopathological progression within 12 months as a clinically actionable endpoint, we acknowledge that our model was not designed or validated to predict longer-term outcomes such as metastasis, treatment escalation, or overall survival. Therefore, its predictive value beyond the first year remains uncertain. Future studies with extended follow-up and specifically tailored models would be required to assess the utility of radiomic features for longer-term disease progression. Additionally, it should be noted that the segmentation networks were trained on imaging data from RP patients with more aggressive cancer than AS patients. This naturally results in larger size and/or number and/or Gleason Score of the lesions, introducing a domain shift between the training and evaluation cohorts. Nevertheless, imaging for both patient cohorts was acquired within the University of Miami Health System, which encompasses a heterogeneous set of MRI vendors and magnet types, providing comparable acquisition protocols and imaging quality across the two groups. The segmentation performance was validated on a subset of the AS cohort and deemed satisfactory after comparisons with similar studies. The combination of Leave-One-Out cross-validation and extensive feature selection may introduce a risk of optimistic performance estimates, as small variations in the training data can influence feature selection and model outcomes. To assess model stability, we examined performance across multiple feature subsets and observed consistent predictive trends, suggesting robustness to variations in feature selection. Nevertheless, external validation in larger, independent cohorts is required to fully evaluate model generalizability and the reproducibility of selected features.

## 5. Conclusions

By integrating multiple pipelines for automatic PCa risk assessment, the risk estimation platform MHP-12mo achieved an AUC of 0.84 in differentiating suitable AS candidates with stable disease from patients who are likely to progress rapidly based on confirmatory mpMRI data and clinical predictor variables from a prospective clinical trial. Intratumoral radiomic features were found to add substantial value to the classification performance, confirming the hypothesis that imaging biomarkers play an important role in progression risk assessment. Albeit in a small cohort, MHP-12mo demonstrated potential to improve PCa risk predictions compared to current standard-of-care protocols.

## Figures and Tables

**Table 1:** Characteristics of the MAST participants at the confirmatory exam. *p*-values computed through *t*-test statistics for continuous variables and chi-square test for categorical variables.

Variable	Training Set *n* (%)	Test Set *n* (%)	*p*
Patients	163	16	
Age, years, median [range, IQR, SD]	62 [43–82, 57–69, 8.25]	62 [51–78, 57.5–67, 7.27]	0.765
Race/Ethnicity			
Non-Hispanic White	80 (49.1)	9 (56.3)	0.921
Hispanic/Latino	67 (41.1)	6 (37.5)	
Non-Hispanic Black	14 (8.6)	1 (6.2)	
Asian	2 (1.2)	0 (0)	
PSA, ng/mL, median [range, IQR, SD]	5.1 [0.5–18.7, 3.8–7.1, 3.1]	5.4 [2–9.4, 4.2–6.4, 2]	0.592
Grade Group			
Benign	48 (29.5)	1 (6.2)	
1	76 (46.6)	15 (93.8)	
2	18 (11.0)	0 (0)	0.011
3	9 (5.5)	0 (0)	
4–5	12 (7.5)	0 (0)	
PI-RADS v2.1			
Negative/1–2	31 (19.0)	3 (18.8)	0.538
3	45 (27.6)	6 (37.5)	
4	70 (42.9)	7 (43.7)	
5	17 (10.4)	0 (0)	
Prostate volume, mL, median [range, IQR, SD]	44.5 [13–143.6, 32.4–61.8, 24.7]	42.0 [19.8–92.3, 40.2–49, 20]	0.797
Treatment (progressors)	87	16	
Prostatectomy	50 (57.5)	9 (56.3)	0.814
Continue AS off protocol	13 (14.9)	2 (12.5)	
Radiation +/− ADT	10 (11.5)	1 (6.2)	
HIFU/Other focal therapy	6 (6.9)	1 (6.2)	
Other/Unknown	8 (9.2)	3 (18.8)	

Abbreviations: IQR = Interquartile Range, SD = Standard Deviation, PSA = Prostate-Specific Antigen, PI-RADS = Prostate Imaging and Reporting Data System.

**Table 2:** Hazard ratios from Cox proportional hazard regression for the DL-based and intratumoral radiomic predictors selected for the progression risk prediction model MHP-12mo.

Feature	Hazard Ratio [95% CI]	*p*-Value
DL segmented prostate volume	0.38 [0.25, 0.57]	<0.001
ADC 10th percentile	0.50 [0.33, 0.77]	0.002
ADC kurtosis	0.58 [0.37, 0.90]	0.014
T2w minimum	0.60 [0.39, 0.91]	0.017
High b-value DWI skewness	1.62 [1.06, 2.47]	0.026

Abbreviations: CI = confidence interval.

**Table 3:** Results of utilizing the risk estimation model MHP-12mo on the 16 patient test set, with the ground truth based on the Adjusted time of progression from the retrospective imaging re-evaluation of these patients. Correct model classifications are marked in bold.

Patient	Time of Progression (Months)	Adjusted Time of Progression (Months)	MHP-12mo Prediction
1	24	24	**Slow progressor (TN)**
2	24	24	**Slow progressor (TN)**
3	36	**12**	**Rapid progressor (TP)**
4	24	24	Rapid progressor (FP)
5	24	24	**Slow progressor (TN)**
6	36	**12**	Slow progressor (FN)
7	24	**0**	**Rapid progressor (TP)**
8	24	**12**	**Rapid progressor (TP)**
9	24	24	Rapid progressor (FP)
10	24	24	**Slow progressor (TN)**
11	24	**12**	**Rapid progressor (TP)**
12	24	**0**	Slow progressor (FN)
13	24	24	Rapid progressor (FP)
14	24	24	**Slow progressor (TN)**
15	24	**0**	Slow progressor (FN)
16	24	24	**Slow progressor (TN)**

Abbreviations: TP = True Positive, FP = False Positive, TN = True Negative, FN = False Negative.

**Figure A1:**
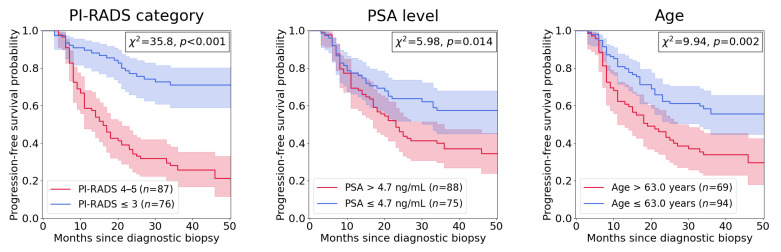
Kaplan–Meier plots for the clinical covariates, with the patient population stratified into two cohorts: a faster progressing cohort shown in red and a slower progressing cohort shown in blue, both with corresponding confidence intervals. Differences in progression-free survival probability between the cohorts were assessed via log-rank test statistics (*χ*^2^).

## Data Availability

The data presented in this study are available on request from the corresponding author.

## Supplementary Materials

The following supporting information can be downloaded at: https://www.mdpi.com/article/10.3390/cancers18050842/s1, Table S1: Summary of confirmatory multiparametric MRI acquisition parameters for the MAST participants.

## Abbreviations

The following abbreviations are used in this manuscript:

ADC	Apparent Diffusion Coefficient
AI	Artificial Intelligence
AS	Active Surveillance
AUC	Area Under (the Receiver Operating Characteristic) Curve
BVAL	Highest b-value Diffusion-Weighted Imaging sequence
CI	Confidence Interval
DCE	Dynamic Contrast Enhanced
DL	Deep Learning
DSC	Dice Similarity Coefficient
DWI	Diffusion-Weighted Imaging
GG	Grade Group
GU	Genitourinary
IRB	Institutional Review Board
IQR	Interquartile Range
MAST	MRI-Guided Active Selection for Treatment of Prostate Cancer
MSRS	Maximally Selected Rank Statistics
mpMRI	Multiparametric Magnetic Resonance Imaging
NPV	Negative Predictive Value
QoL	Quality of Life
PCa	Prostate Cancer
PI-RADS	Prostate Imaging and Reporting Data System
PPV	Positive Predictive Value
PSA	Prostate-Specific Antigen
RP	Radical Prostatectomy
SD	Standard Deviation
T2w	T2-weighted
US	Ultrasound

## Appendix A. Analysis of Clinical Model Features

Kaplan–Meier curves for the clinical predictor variables are shown in Figure A1. The patient population was stratified into a faster progressing cohort and a slower progressing cohort, where the split between the two cohorts was determined by maximally selected rank statistics (MSRS) for each feature. The significant differences in progression-free survival probability between the two cohorts confirm the association of each feature with progression.

Further, applying Cox proportional hazard regression [24] to compute hazard ratios (Table A1) indicated the following significant associations between the various clinical conditions at confirmatory and the likelihood of progression at any point in time:

• A patient with PI-RADS 4 or 5 as the highest assessment category is more than four times more likely to progress than a patient with PI-RADS 3 or below
• A patient with a PSA level above 4.7 ng/mL is 1.7 times more likely to progress than a patient with lower PSA
• A patient above 63 years is close to twice as likely to progress than a younger patient

**Table A1:** Hazard ratios from Cox proportional hazard regression for the clinical predictor variables.

Feature	Hazard Ratio [95% CI]	*p*-Value
PI-RADS category	4.08 [2.48, 6.69]	<0.001
PSA level	1.72 [1.11, 2.67]	0.015
Age	1.95 [1.28, 2.99]	0.002

Abbreviations: CI = confidence interval.

This analysis confirms the three clinical variables incorporated into the progression risk model to be associated with increased risk of quick disease progression, something which is already well-studied. Patients with higher PI-RADS scores are more likely to show signs of cancer progression [51] and a significantly elevated PSA level is generally associated with a higher likelihood of aggressive PCa [52]. Meanwhile, our study found PSA to be a less strong predictor which might stem from the fact that patients enrolled in the AS trial must have a PSA level elevated enough for concern of PCa yet relatively low (≤20 ng/mL), resulting in the spectrum of PSA levels among the particular patient population under study being quite narrow. Increased age was shown to impact progression, in line with evidence that older patients have a higher risk of being affected by more aggressive and fast-progressing PCa compared to younger patients [53].
