# Supplementary material for: mpMRI-Based Risk Estimation to Optimize Prostate Cancer Patient Selection for Active Surveillance
Source: Cancers (Basel). 2026 Mar 5;18(5):842. doi: 10.3390/cancers18050842 (PMC12984811; doi:10.3390/cancers18050842)
Supplement: Supplementary file 1 [file cancers-18-00842-s001.zip › cancers-4149409-supplementary.pdf]

Table S1. Summary of confirmatory multiparametric MRI acquisition parameters for the MAST participants.

		**GE Discovery MR750 (Waukesha, WI)** *n*=37	**Siemens Skyra (Erlagen, Germany)** *n*=82	**Siemens TrioTim (Erlagen, Germany)** *n*=43	**Siemens Vida (Erlagen, Germany)** *n*=10	**Siemens Avanto (Erlagen, Germany)** *n*=7
**T2w**	Matrix size (pixels)	512×512×(23,24,26,27,29), 256×256×(25,72)	512×512×(32,72), 320×320×(32,34) , 464×512×(64,72), 416×512×(64,72), 384×512×72, 384×384×(30,32), 400×512×72, 432×512×64	384×512×72, 512×512×(25,27,29,35,65,72), 464×512×72, 320×320×65, 384×512×70	512×464×72, 320×320×32	320×320×(23,24), 176×256×35, 192×256×100
	Voxel size (mm^3)	0.43×0.43×3.0, 0.391×0.391×3.0, 1.25×1.25×2.5, 0.684×0.684×2.5, 0.781×0.781×3.0	0.703×0.703×2.5, 0.625×0.625×3.0, 0.391×0.391×3.0, 0.688×0.688×3.0, 0.521×0.521×3.0	0.703×0.703×2.5, 0.43×0.43×3.0, 0.391×0.391×3.0, 0.508×0.508×3.0, 0.586×0.586×2.5, 0.625×0.625×3.0	0.703×0.703×2.5, 0.688×0.688×3.0	0.625×0.625×4.0, 1.562×1.562×6.0, 1.562×1.562×2.5
	TR (ms) Min-Max	3800-10998	3380-6270	3200-8920	3700-6100	3400-10720
	TE (ms) Min-Max	80.65-108.1	89-114	82-122	89-114	92-96
	ETL	16, 21	21, 23	21,23	21, 23	25, 29
**ADC**	Matrix size (pixels)	256×256× (26,35,36,39,40)	96×128× (36,38,80), 120×160× (24,28,30,35,36,38,40,50), 122×160×38	96×128×38 98×130×38	120×160×38	144×192×35 138×192×35 192×222×35
	Voxel size (mm^3)	1.25×1.25×2.5	2.5×2.5×2.5, 2.93×2.93×2.5, 2.0×2.0×2.5, 2.344×2.344×2.5, 1.562×1.562×2.5, 1.562×1.562×5.0	2.93×2.93×2.5, 3.125×3.125×2.5, 1.938×1.938×2.0, 2.344×2.344×2.5, 1.953×1.953×5.0, 2.023×2.023×5.0	2.344×2.344×2.5, 1.562×1.562×5.0	2.083×2.083×7.0, 2.188×2.188×7.0, 1.802×1.802×2.5, 1.979×1.979×.7.0
	TR (ms) Min-Max	8500-9500	5170-9500	5200-5800	7300	6000-7300
	TE (ms) Min-Max	52.4-59.3	64.0-93.0	95.0-104.0	61.2-64.0	72.0-84.0
	DWI b-value sequence (s/mm^2)	50-500-1000, 50-500-1000-1400	50-500-1000, 50-500-1400, 50-1000-2000, 50-500-1000-1400	50-1000-2000, 50-500-1000-1400	50-500-1000-1400	50-500-1000-1400
**DCE**	Matrix size (pixels)	256×256×(36,38,72)	288×320×(40,52) 384×512×72, 320×320×40	160×160×40, 384×512×72	288×320×40	272×320×40
	Voxel size (mm^3)	1.25×1.25×2.5, 1.016×1.016×2.5, 1.117×1.117×2.5	0.812×0.812×2.5, 0.703×0.703×2.5, 0.875×0.875×2.5, 0.844×0.844×2.5	1.625×1.625×2.5, 1.375×1.375×2.5, 1.5×1.5×2.5, 0.703×0.703×2.5, 1.438×1.438×2.5, 1.938×1.938×2.5	0.812×0.812×2.5, 0.875×0.875×2.5	0.875×0.875×2.5, 1.125×1.125×2.5
	TR (ms) Min-Max	3.04-4.06	4.36-5.24	3.76-5.08	4.36	4.09
	TE (ms) Min-Max	1.36-1.78	1.90-2.33	1.28-2.30	1.90-1.92	1.64-1.74
	Timepoints	12, 48	12, 48	12, 48	48	47
	Temporal resolution (s) Min-Max	7.5-31.29	7.5-31.5	7.5-34.0	7.5	10.5-11.5
